# Associations between wearables vital parameters and self-perceived mood—an ecological momentary assessment study among healthy adolescents

**DOI:** 10.3389/fpsyg.2025.1623886

**Published:** 2025-12-24

**Authors:** Luisa Lutz, Polina Veltmann, Romain Meisterhans, Marco Giurgiu

**Affiliations:** 1 Department of Sport and Exercise Science, Faculty of Natural and Life Sciences, Paris Lodron University of Salzburg, Salzburg, Austria; 2 Smilamind AG, St. Gallen, Switzerland; 3 Institute of Sports and Sports Science, Karlsruhe Institute of Technology (KIT), Karlsruhe, Germany

**Keywords:** ecological momentary assessment, ambulatory assessment, mood, mental health, physical behavior, accelerometry, adolescents

## Abstract

**Background:**

The prevalence of mental illnesses among adolescents has increased over the past decade. Lifestyle factors, such as regular exercise or sleep behavior can play a significant role in the prevalence of mental health. Today, passive mobile sensing or smartwatches offer valuable insights into the relationships between device-derived vital parameters (e.g., steps, exercise, sleep, heart rate) and self-reported mental health indicators, such as mood. However, the link between vital parameters and self-reported mood in daily life among adolescents remains understudied.

**Methods:**

A total of 53 adolescents participated in a two-week ambulatory assessment study. Participants were equipped with a wrist-worn Apple Watch and a research-grade thigh-worn accelerometer to continuously measure physical behavior in daily life. Concurrently, participants rated momentary mood up to six times a day on a self-developed mood barometer (i.e., a 10-point emoji scale) via a web-based application on a smartphone. We used multilevel modeling to analyze the within-subject effects of vital parameters on momentary mood.

**Results:**

Step counts (*p* = 0.023), standing time (*p* = <0.001), as well as exercise time (*p* = 0.012) are positively associated with self-reported mood on a daily level. On a momentary level, step counts (*p* = 0.006) before the assessment (60 min) were positively associated with momentary mood, whereas sedentary time (*p* = <0.001) and sedentary bouts (*p* = 0.003) were negatively associated. Passively detected sleep duration was not associated with daily mood, whereas self-reported sleep quality (*p* = 0.031) was positively associated.

**Conclusion:**

Our study indicates that vital parameters correlate with mood ratings on both momentary and daily levels in adolescents. Given the increasing prevalence of mental health disorders, there is an urgent need for practical, scalable solutions. Mobile health technologies designed to enhance mental health support for adolescents and younger adults show significant promise, making them a compelling focus for future research.

## 1 Introduction

Mental health disorders represent a significant global burden, accounting for 15% of all diseases among adolescents aged 10 to 19 in terms of years lived with disability. Among these, anxiety (4.6%) and depression (2.8%) are the most prevalent (Rehm and Shield, 2019; World Health Organization, 2024b). According to the World Health Organization (WHO), mental, behavioral, and neurodevelopmental disorders involve considerable disruptions in cognition, emotional regulation, or behavior, typically stemming from underlying psychological, biological, or developmental dysfunctions (World Health Organization, 2024a). The overall prevalence of these disorders has increased over the past two decades (Krokstad et al., 2022; Yang et al., 2024). Experiencing mental illness during adolescence also increases the likelihood of these conditions persisting into adulthood (Sawyer et al., 2018). The COVID-19 pandemic further exacerbated mental health challenges by disrupting adolescents’ daily lives—reducing social interaction, limiting leisure activities, and changing physical activity patterns (Pfefferbaum and North, 2020). These disruptions contributed to a rise in mental health issues, particularly anxiety and depression (GBD 2019 Mental Disorders Collaborators, 2022; Wu et al., 2023).

Determinants of mental disorders encompass a wide range of factors, including socio-economic status, education, exposure to trauma, genetic predisposition, and overall environment (World Health Organization, 2014; Furber et al., 2017). While many of these factors are difficult to change, a key element in preventing mental disorders lies in the adoption of modifiable lifestyle behaviors—such as smoking, nutrition, sleep behavior and physical activity (PA; Atzendorf et al., 2018). For instance, a previous study has shown, that using a clustering algorithm to classify adults by lifestyle patterns found a strong link between health risk behaviors—such as alcohol use, low PA, and smoking—and the presence of mental disorders (Vermeulen-Smit et al., 2015). Notably, these modifiable lifestyle behaviors often shift during the transition from adolescence to adulthood (e.g., dropping out of organized sports; Crane and Temple, 2015). Research has shown that avoiding drug and substance use, maintaining good sleep quality and duration (typically 6–9 h), consuming a healthy diet (e.g., rich in fruits and vegetables, low in fat) (Owen and Corfe, 2017), minimizing sedentary behavior (SB), and engaging in regular PA all contribute to the prevention of depression in adolescents and young adults. Among these factors, PA—defined as any bodily movement produced by skeletal muscles that results in energy expenditure (Caspersen et al., 1985)—has been widely studied for its positive impact on mental health. It is associated with improved mood and emotional states in daily life (Ivarsson et al., 2021), reduced symptoms of acute depressive disorders (Goodwin, 2003; Todder et al., 2009; Cao et al., 2020), and is also recognized as an effective therapeutic intervention for various mental health conditions (Todder et al., 2009). Conversely, a sedentary lifestyle has been linked to poorer mental health outcomes and may elevate the risk of developing depression (Teychenne et al., 2010; Hallgren et al., 2020a). A recent health survey spanning 68 countries underscores the widespread prevalence of SB and mental health symptoms among adolescents, revealing a clear dose–response relationship between sedentary time and adverse mental health outcomes (Shawon et al., 2025).

Sleep, completing the 24-h day cycle, is widely recognized as a key health-related behavior. It is a naturally recurring and easily reversible state, marked by reduced or absent consciousness, perceptual disengagement, immobility, and the adoption of a typical sleep posture. According to the Consensus Statement of the American Academy of Sleep Medicine and the Sleep Research Society, sleep plays a vital role in numerous aspects of human health (Watson et al., 2015). Sleep is critically involved in systemic physiological processes, including metabolism (Magee and Hale, 2012), cardiovascular function (Wang et al., 2012), mood regulation (Minkel et al., 2012), and brain functions such as neurobehavioral performance, cognitive processing, and safety-related tasks (van Dongen et al., 2003). Additionally, sleep influences a wide range of other health outcomes (Watson et al., 2015).

Smartphones and smartwatches offer a cost-effective and efficient way to monitor lifestyle and health factors in everyday life. In Germany, more than 90% of adolescents (ages 12–19) own a smartphone, and one-third regularly use wearable devices (Feierabend et al., 2024). This widespread use makes smartphones and wearables valuable tools for both passive and active data collection, enabling the tracking of lifestyle behaviors, vital signs, and early indicators of clinically relevant changes—such as shifts in movement patterns over time (Mohr et al., 2017). Adolescents and young adults, in particular, are well-suited for behavior monitoring through smartphone applications (Mohr et al., 2017; Meegahapola and Gatica-Perez, 2021; Marciano et al., 2023) and often show a pragmatic but open-minded attitude toward mental health apps (Götzl et al., 2022). Dubad and colleagues also emphasize the potential of app-based self-monitoring for assessing specific mental health disorders (Dubad et al., 2018). One key advantage of commercial wearables over research-grade devices is their seamless integration into daily life, as users are not required to carry additional equipment—these devices are already part of their routine (Blaauw et al., 2016; Russell and Gajos, 2020). However, challenges should be acknowledged such as data privacy concerns, technological barriers for certain populations, or the digital divide that may impact technology access among adolescents.

In the research community, a suitable methodology for capturing physical behavior and psychological variables as well as their changes in real-life settings is ambulatory assessment (AA; Reichert et al., 2020). AA enables time-sensitive measurement of psychological variables through repeated self-monitoring (e.g., electronic diaries (e-diaries) on smartphones) and can be combined with device-based measurements using wearables (Fahrenberg et al., 2007; Kanning et al., 2013). AA is used to collect multiple assessment points within participants (e.g., several mood ratings per person per day) and allows researchers to observe dynamic processes and gain insights into contextual circumstances (Fahrenberg et al., 2007; Ebner-Priemer and Trull, 2009). Focusing on capturing mood or emotions, AA comes along with the advantage of collecting data in real-time and avoiding recall biases (Fahrenberg et al., 2007; Kanning et al., 2013; Kaufmann et al., 2020; Russell and Gajos, 2020). Mood, emotion, and affect are often used as synonyms and are defined with inconsistent concepts (Williams et al., 2018). While emotions have specific stimuli and appraisal that lead to different physical and psychological responses (Lazarus, 1991; Williams et al., 2018), moods are more diffuse than emotions and can occur over longer periods, they involve a change in cognitive appraisal, a behavior change, and core affect as do emotions (Williams et al., 2018). In contrast, core affect is defined as a non-reflective simple feeling that does not require cognitive appraisal and can range from positive to negative and vary in activation (low to high; Russell, 2003; Williams et al., 2018).

A recent systematic review of ambulatory assessment (AA) studies has highlighted a reciprocal relationship between PA and mood in adolescents and young adults (Timm et al., 2024). Overall, within-subject analyses show that higher levels of PA are positively associated with enhanced mood and increased positive affect across children, adolescents, and adults (Timm et al., 2024). These findings are echoed by Bourke et al., who reviewed studies focused specifically on children and adolescents and also reported a positive within-person association between PA and mood (Bourke et al., 2021a). However, when examining different types and intensities of PA in everyday life, research has produced mixed results regarding its influence on subsequent mood (Koch et al., 2020; Bourke et al., 2023). Beyond PA, SB has also been linked to mood: adolescents tend to report higher levels of negative affect during periods when they are more sedentary than usual (Cushing et al., 2017). For instance, sedentary bouts lasting 30 min or longer have been shown to negatively affect both valence and energetic arousal in samples of university employees (Giurgiu et al., 2019). In children, however, context-adjusted sedentary time in the 30 min following momentary assessments was not significantly associated with positive affect (Kracht et al., 2021). In addition to activity levels, other vital parameters captured by wearable devices—such as sleep parameters and heart rate—also show connections to mood. Variability in heart rate (HR), as measured in AA studies, has been linked to both valence (Exler et al., 2016) and positive affect (Hachenberger et al., 2023). Both self-rated sleep duration and sleep quality have been found to be linked to next day mood, with a significantly stronger associations for sleep quality in an adolescent group (Shen et al., 2018). Hickman et al. concluded that self-rated subjective sleep markers are stronger associated to mood and affect than objective sleep markers (Hickman et al., 2024). Short et al. revived sleep durations in adolescents and reported a higher risk for lower mood in individuals with short sleep (Short et al., 2020). Shen and colleagues analyzed the bidirectional sleep–affect associations while differentiating between- and within-person effects (Shen et al., 2022). The authors concluded that overall positive affect may have a protective impact on adolescent sleep duration (Shen et al., 2022).

A review by Timm et al. highlights a significant research gap regarding the relationship between physical behavior and mood in adolescents, with only 11 of 66 studies focusing on this group—nine on healthy adolescents—and just two exceeding 1 week in duration (Timm et al., 2024). Further research is needed to clarify the specific components linking PA to mood in adolescents, especially given the inconsistent findings on exercise versus non-exercise activity and SB to mood (Bossmann et al., 2013; Koch et al., 2020; Bourke et al., 2023). Based on previous empirical studies (Bossmann et al., 2013; Exler et al., 2016; Cushing et al., 2017; Koch et al., 2018; Giurgiu et al., 2019; Bourke et al., 2021b; Bourke et al., 2021a; Bourke et al., 2023; Kracht et al., 2021; MacLeod et al., 2021; Simon et al., 2021; Yang et al., 2021; Giurgiu and Ebner-Priemer, 2023; Hachenberger et al., 2023; Do et al., 2024; Timm et al., 2024), we hypothesize that on a day level basis (1a) PA (i.e., higher step count) positively predicts self-perceived mood; (1b) Standing time positively predicts self-perceived mood; (1c) Exercise time positively predicts self-perceived mood. Furthermore, we hypothesize that on a momentary level (2a) PA (i.e., higher step count) 60-min prior to the assessment positively predicts self-perceived mood; (2b) SB 60-min prior to the assessment negatively predicts self-perceived mood; (2c) Sedentary bouts (i.e., 30-min) negatively predict self-perceived mood. Furthermore, we conducted exploratory analyses to gain insights between vital parameters measured by wearables (i.e., heart rate, sleep duration, and sleep quality) and self-perceived mood. As an additional robustness check, we replicated the models with an established mood measure (MDBF short scale; Wilhelm and Schoebi, 2007).

## 2 Methods

### 2.1 Participants

Between November 2023 and May 2024, we recruited 53 adolescents and young adults. The sample included both university and school students and individuals who are employed. We included only participants aged between 13 and 20 years, who were able to perform daily life activities, meaning they were currently not diagnosed with any physical injuries or mental diseases. In total, 4 participants were excluded from the analyses because either they were not compliant in answering momentary mood or did not fulfill the wear time criteria of the used wearables. Thus, the final sample consisted of 49 participants (65.3% females) with a mean age of 17.88 ± 1.93 years and a mean body mass index (BMI) of 21.88 ± 2.93 kg/m^2^. Data was collected anonymously, and the study fully conformed to the Declaration of Helsinki and the ethics guidelines of the German Psychological Society [BDP (Berufsverband Deutscher Psychologinnen und Psychologen e.V.), and DGPs (Deutsche Gesellschaft für Psychologie), 2016]. Participants received detailed information regarding voluntary participation, the handling of the devices and questionnaire, and the processing of their data, and they gave written informed consent according to the ethics guidelines of the German Psychological Society [BDP (Berufsverband Deutscher Psychologinnen und Psychologen e.V.), and DGPs (Deutsche Gesellschaft für Psychologie), 2016]. For participants younger than 18 years of age, consent was obtained from their parents. According to the guidelines of the ethics committee of the Karlsruhe Institute of Technology (KIT), the German Research Foundation [DFG (Deutsche Forschungsgesellschaft), 2019], and the National Science Foundation [NSF (U.S. National Science Foundation), 2019], the study was exempt from the institutional ethics committee review because it was purely observational (noninvasive) and did not induce any type of psychological stress or anxiety. Participants were free to withdraw from the study at any time. Participants received an online voucher worth €50 as an incentive to take part.

### 2.2 Study procedures

**Figure 1:**
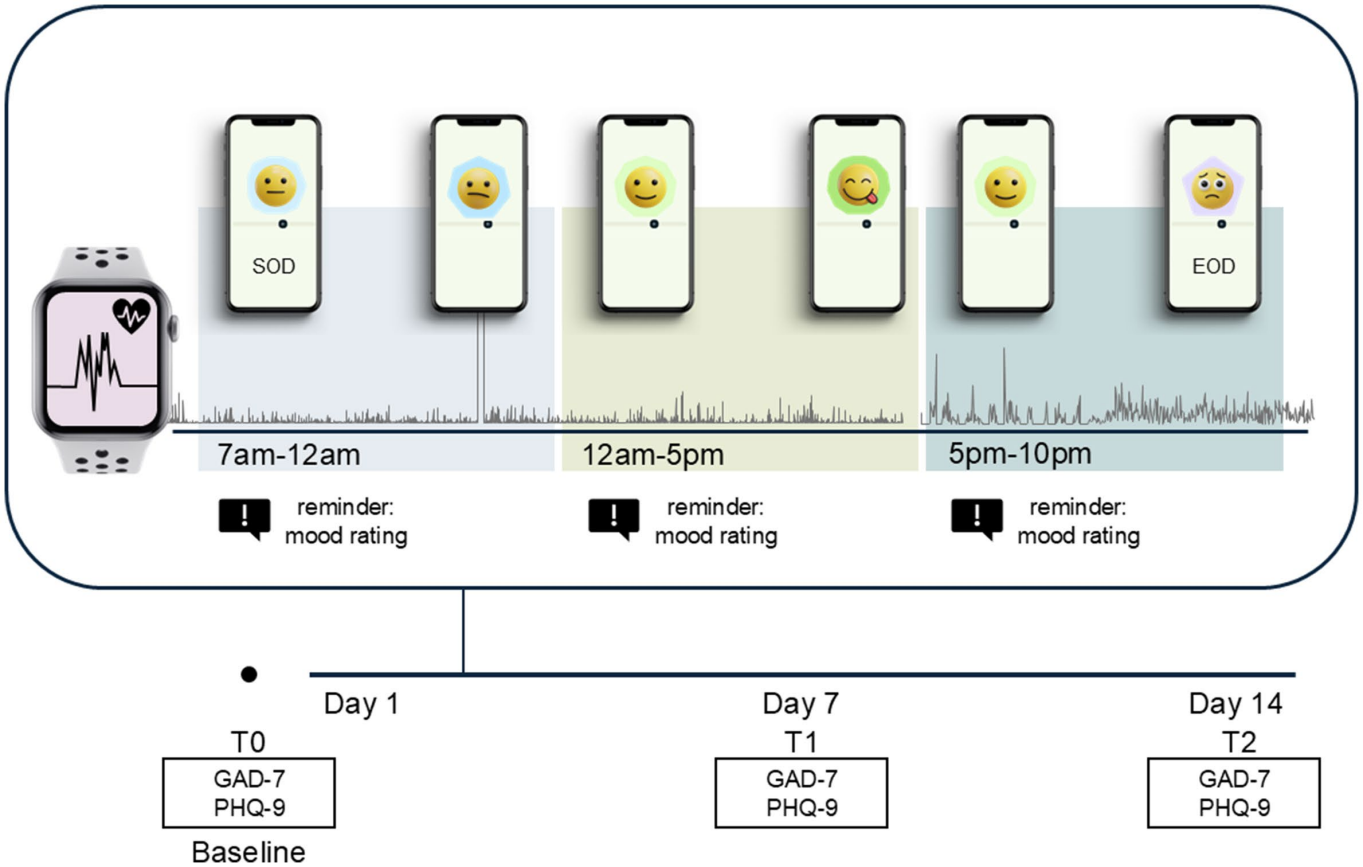
Overview of the study procedure. All emojis designed by OpenMoji - the open-source emoji and icon project. License: CC BY-SA 4.0.

After recruiting via flyers, and word of mouth, participants completed an initial in-person session. During this appointment, participants received written and oral information regarding the study procedures, were trained with the technical equipment, and filled in questionnaires. After the in-person session, participants started with the AA study for 14 days. The link to the Smilamind web-based portal was added to the home screen of their smartphone and the participants received personal access to log into the portal. Participants were equipped and instructed to wear the Apple Watch SE Generation 2 (Apple Inc., California, United States, apple.com) on the wrist continuously for 24 h per day. Additionally, participants received the research-grade accelerometer move 4 (movisens GmbH, Karlsruhe, Germany, movisens.com) and were encouraged to wear the device on their right thigh. The primary rationale for using both sensor systems was to facilitate a comparative analysis of the associations between physical activity and mood, leveraging disparate algorithms. Additionally, the objective was to transfer the methodologies and findings derived from the study into everyday practice, a task for which the utilized commercial tracker plays a pivotal role. A further technical consideration was that the Apple Watch provides all required data streams and offers a well-documented and robust software development kit. Each day between 7 a.m. and 10 p.m., the participants were able to report their current momentary mood as well as contextual factors. The participants were free to choose the rating time. To receive a balanced distribution of momentary ratings across the day, we encouraged the participants to rate their mood twice between 7 a.m. to 12 p.m.; 12 p.m. to 5 p.m.; and 5 p.m. to 10 p.m., respectively. The participants received a short reminder on their Apple Watch three times a day to ensure they did not forget to rate their mood. One assessment point was finalized after completing the momentary ratings, which lasted approx. 90 s. In addition to the momentary ratings, the participants were instructed to complete psychological questionnaires (i.e., GAD-7, PHQ-9) in the web-based portal at the beginning, after 7 days, and after 14 days. Following the completion of the study, participants had a further in-person session where they returned the study equipment. The full study procedure is visualized in Figure 1.

### 2.3 Smilamind application

**Figure 2:**
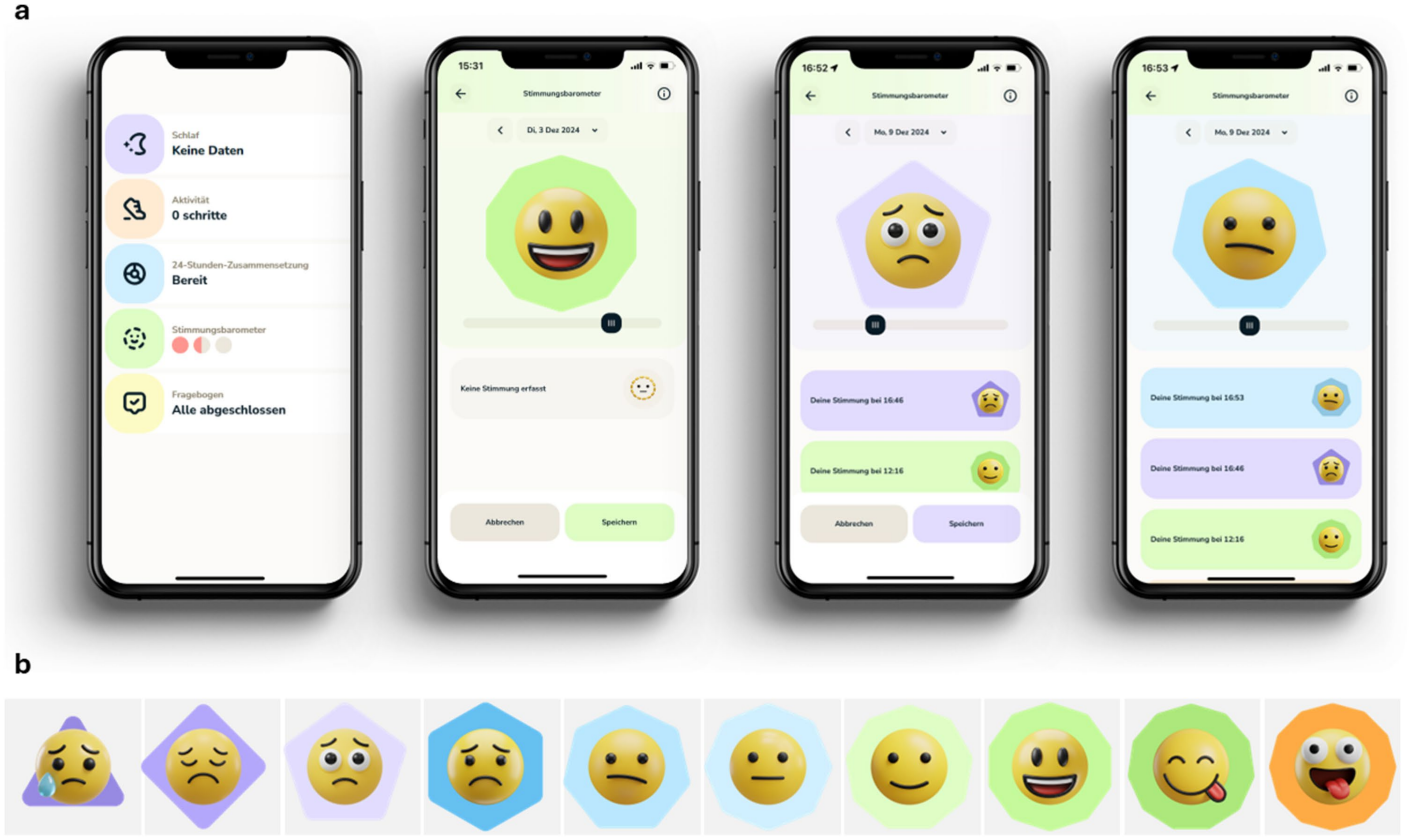
**(a)** Insights in the Smilamind web-based application (overview and mood rating through the day). **(b)** Emoji-scale for mood rating, lowest to highest mood. All emojis designed by OpenMoji - the open-source emoji and icon project. License: CC BY-SA 4.0.

The Smilamind web-based application (Smilamind AG, St. Gallen)^1^ is a self-developed mobile health solution intended to digitally enhance mental health services for children, adolescents, and younger adults. The application in the presented study comprises three key elements, i.e., a software solution with a technical interface and connection to wearables vital parameters. First, we used a web-based portal where participants received individual accounts for registration and access to report data (e.g., momentary mood ratings). A study coordinator used the web-based portal to monitor compliance and download all data. Second, we provided Apple Watch devices to all participants. On each device, a self-developed Apple Watch application was manually deployed. In detail, selected vital parameters were stored on the watch and can be uploaded to the application programming interface (API) by the participants via the Apple watch application. Third, the API is used in the background to collect, assign, and display data from various sources (i.e., wearable and self-reported data). Examples of the application are illustrated in Figure 2.

### 2.4 Measures

#### 2.4.1 Vital parameters derived from wearables

The Apple Watch is a commercial smartwatch including a variety of different sensors (e.g., accelerometer, optical heart sensor, gyroscope, and ambient light sensor; Apple Inc., 2024a). The Apple Watch runs with the watchOS 10.1. Software, Health Kit was used to access the health data on the watch. The Apple Watch provides a variety of following vital parameters: (i) *Exercise time* counts the minutes when the user reaches the intensity of fast walking, which is adjusted to the users’ fitness level through proprietary algorithms; (ii) *Standing time* counts the minutes users stand or walk; (iii) *Heart rate* is measured periodically throughout the day (i.e., when no movement occurs at irregular intervals, approximately every 10 min and continuously when a workout is detected) using photoplethysmography, a green LED paired with light-sensitive sensors that can measure a range of 30–210 beats per minute; (iv) *Sleep duration* and *sleep states* are captured by movement data; (v) *Step count* is measured by the accelerometer in the device (Nelson and Allen, 2019; Apple Inc., 2023; Apple Inc., 2024b). Previous studies indicated mixed findings regarding the validity of the vital parameters provided by the Apple Watch (Bai et al., 2018; Fuller et al., 2020). The move 4 accelerometer captured movement and non-movement behaviors with a range of ± 16 g and a sampling frequency of 64 Hz. Raw acceleration data were stored on an internal memory card and were processed by a band-pass filter (0.25–11 Hz) to eliminate artifacts. Previous validation studies have shown that the move accelerometer is appropriate to differentiate between sitting/lying and upright body postures (Giurgiu et al., 2020), and to capture intensity levels of PA (Anastasopoulou et al., 2012). To parameterize the accelerometer values, we calculated relevant parameters (i.e., body position and movement acceleration intensity [millig]) in 1-min intervals by using the software DataAnalyzer (version 1.16.8; movisens.com).

#### 2.4.2 Momentary mood ratings

To assess momentary mood, we used a self-developed single-item emoji scale to capture the age-appropriate intuitive recording of affective states. In particular, the scale included 10 different emojis (see Figure 2b). In addition, we used a validated short scale consisting of six items (Wilhelm and Schoebi, 2007). The scale captures three basic affective state dimensions, including valence (V), energetic arousal (EA), and calmness (C). The six bipolar items were presented to participants in German translation on visual analog scales (0–100). The items include: EA1—tired to awake; V1—content to discontent; C1—agitated to calm; EA2—full energy to without energy; V2—unwell to well; C2—relaxed to tense.

#### 2.4.3 E-diary ratings

In addition to mood ratings, we assessed cognitive load [scale 0–100] and self-perceived stress (5-point Likert) at each momentary rating. Additionally, at the first assessment point of the day, we asked whether the participants had worn the wearables at night, the number of awake periods through the night, hours of sleep, and self-perceived sleep quality. At the last assessment point of the day, we asked about their eating behavior, pain throughout the day, questions including exercise (i.e., strengthening and other sports), and time spent in nature.

### 2.5 Statistical analyses

Before the statistical analysis, data were removed due to wear time criteria. Contrary to the move accelerometer, wear time parameters are not directly available for Apple Watches through provided data. An indicator of wear time is heart rate (Lin et al., 2020; Golbus et al., 2021; Firkin et al., 2023). HR is measured periodically at rest and more frequently during PA (Nelson and Allen, 2019; Apple Inc., 2023). Thus, non-wear time can be defined by periods without HR recordings. As rough wear-time criteria, we defined any hour with ≥ six HR recordings per hour as a full hour of wear time, whereas hours with less than six heart rate recordings per hour were excluded. A valid day was defined with ≥ 20 h wear time hours counted (Blodgett et al., 2024). Further, participants were excluded from the data set, when they had less than three valid days. The same criteria were used for the move sensor. The merged minute-by-minute values of the Apple Watch data and the e-diary ratings were downloaded from the Smilamind web portal. Afterward, the move 4 data was merged into the data set using the statistic software SPSS (version 28). To analyze our hypotheses, we set up two-level multilevel models as the state-of-the-art procedure for analyzing hierarchically structured intensive longitudinal data (Bolger and Laurenceau, 2013). In particular, we calculated a random intercept random slope model with repeated daily measurements (level 1) nested within participants (level 2). First, intraclass correlations (ICCs) were estimated using unconditional models, including mood parameters (emoji scale, valence, energetic arousal, and calmness) as outcomes. Second, we added the following parameters as predictors: time [hours], time-squared [hours-squared], age [yrs], sex [male vs. female], day [weekend day vs. weekday], BMI [kg/m2], sedentary time [min] exercise time [min], standing time [min], and steps to our models. To test hypothesis 2, we conducted a random intercept random slope model with repeated momentary measurements (level 1) nested within participants (level 2). Compared to hypothesis 1, we added the momentary predictor’s steps count in the 60-min before the mood rating, time spent sedentary in the 60-min before the mood rating [min], and sedentary bouts [bout vs. no bout 30-min before the mood rating]. To standardize time and time-squared, we subtracted the study’s start time for each day (7.30 a.m. on workdays and 9.30 p.m. on weekend days), respectively. According to Arend and Schäfer (2019) rules of thumb for minimum detectable effect sizes (MDES), our data allow the detection of small effects for within-subject associations and medium to large effects for between-subject associations. For exploratory analyses we added HR and sleep duration [h/day], as well as self-rated sleep quality as predictors. We included significant (*p* < 0.05) random effects for each predictor. In interest if model parsimony, nonsignificant random effects were deleted. The equations for the models are presented in the Supplementary file S1. To compare effects between predictors, we calculated standardized beta coefficients (standardized *β*) following established procedures (Hox and Roberts, 2014). All analyses were conducted using SPSS (version 28, IBM). We set the *α* level to 0.05 for all analyses.

## 3 Results

### 3.1 Descriptive results

**Figure 3:**
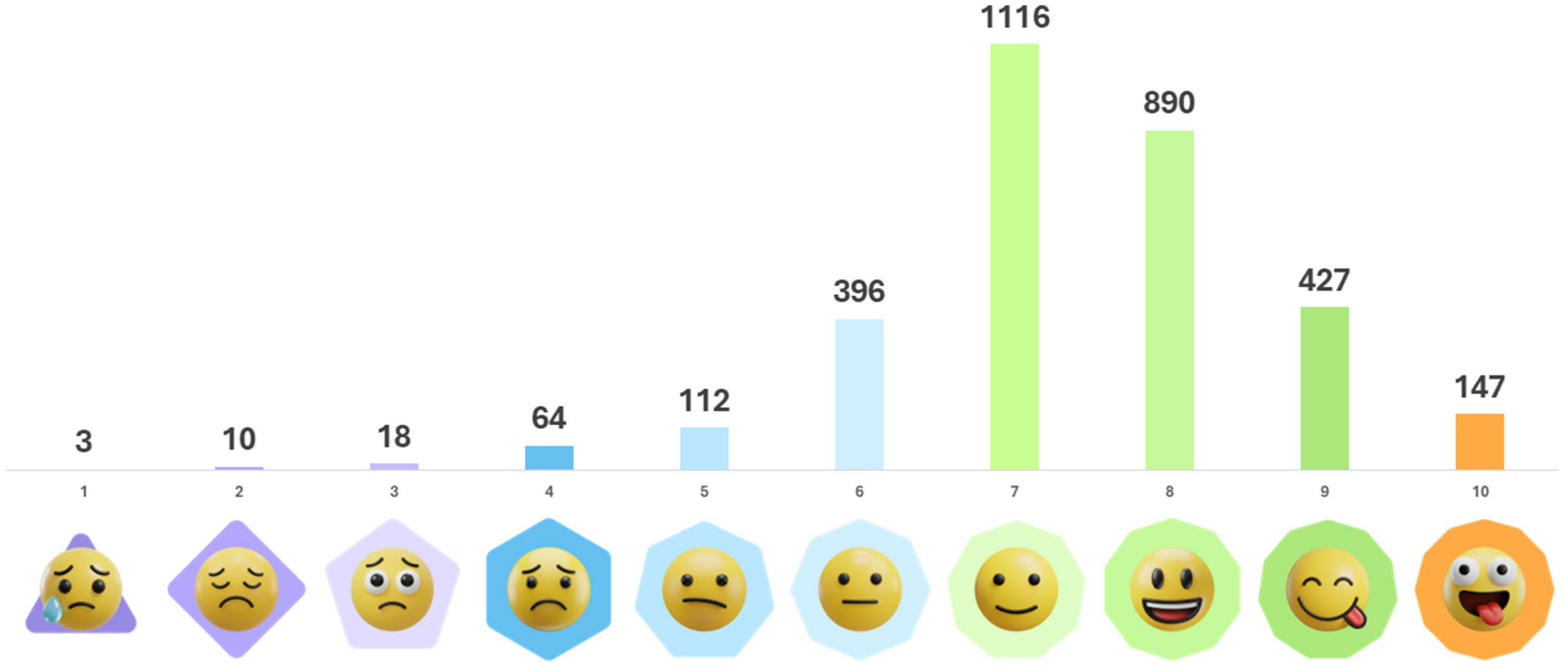
Distribution of total mood ratings across the emoji scale. All emojis designed by OpenMoji - the open-source emoji and icon project. License: CC BY-SA 4.0.

All sample characteristics are presented in Table 1. In total, participants ranked their mood 3,183 times across 14 days (i.e., 4.72 ratings/participant/day on average; ranging from 1.22 to 6.43 (SD = 0.92) on a person level). The average mood score was 7.39. The distribution of the used emoji scales is visualized in Figure 3. The ICC was 0.49, indicating that 51% of the variance in the mood ratings is explained by within-person differences. Average scores for the three basic mood scores are 70.71 (SD = 12.98; valence), 55.89 (SD = 13.91; energetic arousal), and 69.22 (SD = 14.13; calmness). The emoji scale used in the Smilamind Web Application correlates significantly with the three mood dimensions valence (*r* = 0.67, *p* < 0.001), energetic arousal (*r* = 0.56, *p* < 0.001), and calmness (*r* = 0.46, *p* < 0.001).

**Table 1:** Descriptive statistics.

Device	Variables	N, Mean ± SD	Min	Max
	Female (%)	*N* = 32; 65.3%		
	Age (years)	17.88 ± 1.93	13	20
	BMI (kg/m^2^)	21.88 ± 2.94	17.58	32.87
	Emoji scale (1–10)	7.38 ± 0.69	5.67	9.19
	Self-reported sleep quality (0–100)	63.28 ± 15.17	23.00	98.80
Apple watch	Wear Time^1^	22.53 ± 1.22	20	24
	Step count (per day)^3^	8782.34 ± 3578.55	2765.07	23327.02
	Standing time (min)^3^	758.55 ± 80.17	531.43	913.85
	Heart Rate (min)^3^	76.86 ± 7.66	57.15	98.90
	Exercise time (min)^3^	23.73 ± 15.61	3.71	78.85
	Sleep duration (min)^3^	*N* = 48; 426.89 ± 55.05	289.00	544.64
Move 4^2^	Step count (per day)^1^^,^^3^	8730.92 ± 3011.84	3022.40	15529.44
	Sleep duration (min)^1^^,^^3^	*N* = 46; 431.10 ± 89.07	133.50	746.00
	Sedentary time (min)^1^^,^^3^	1115.58 ± 80.84	915.27	1302.45
	Sedentary bouts (≥ 30 min)^1^^,^^3^	*N* = 48; 1.88 ± 0.56	0.60	2.95

1 Based on included data.

2 Data derived from the move 4 sensor based on 50 valid participants.

3 Aggregated values per participant per day.

### 3.2 Association between vital parameters and mood on a daily level

Associations between vital parameters measured by the Apple Watch and self-perceived mood ranked on the emoji Scale on a day level are presented in Table 2. As hypothesized, a higher step count per day positively predicted daily mood (stand. *β* = 0.097; *t* = 2.279; *p* = 0.023). In practice, when taking 5,000 steps per day instead of 1,000 steps per day the predicted daily mood rises from 6.22 to 6.29, and taking 10,000 steps will predict the daily mood with 6.38 (scale 0–10). As assumed (hypothesis 1b), standing time positively predicted daily mood (stand. *β* = 0.131; t = 3.527; *p* < 0.001); that is, spending more time standing was associated with higher mood ratings. More time spent daily with exercise positively predicted daily mood (stand. *β* = 0.111; t = 2.513; *p* = 0.12), supporting hypothesis 1c. For example, spending 120 instead of 10 min exercising increased mood by 0.5 units on average (scale 1–10). Furthermore, the day of the week positively influenced mood (*p* = 0.012). In practice, mood was lower rated on weekdays by 0.16 units compared to weekend days. No significant associations were observed between the time-independent variables age, BMI, sex, and daily mood. Exploratory analyses revealed that higher self-reported sleep quality was associated with higher mood ratings (stand. *β* = 0.611; t = 4.254; *p* = 0.031). Sleep duration in hours and daily HR was not significantly associated with mood. Detailed results are shown in the Supplementary file S2. As an additional analysis to check for robustness, we replicated the reported models while including the affective states measured with the MDBF scale (i.e., valence, energetic arousal, and calmness) as dependent variables (see Supplementary file S3). Those models revealed that more PA was associated with higher valence (stand. *β* = 1.071; *t* = 2.363; *p* = 0.019). Time spent in standing positions is positively associated with valence (stand. *β* = 1.064; *t* = 1.985; *p* = 0.048).

**Table 2:** Multilevel analysis for hypothesis 1.

Type of effect	Outcome	Emoji scale		
	Hypothesis	1a	1b	1c
		b (SE)	b (SE)	b (SE)
Fixed effects	Intercept	6.201 (1.204) **	6.220 (1.204) **	6.199 (1.214) **
	Age (yrs)	0.026 (0.052)	0.026 (0.052)	0.026 (0.053)
	BMI (kg/m^2^)	0.036 (0.033)	0.036 (0.033)	0.035 (0.034)
	Sex^1^	0.197 (0.212)	0.197 (0.212)	0.201 (0.214)
	Weekend^2^	−0.163 (0.065) *	−0.187 (0.065) *	−0.167 (0.066) *
	Step count	1*10^−5^ (2*10^−6^) *	-	-
	Standing time	-	0.001 (2*10^−4^) **	-
	Exercise time	-	-	0.005 (0.002) *

*p < 0.05; **p < 0.001.

1 males compared to females.

2 weekend compared to weekdays.

### 3.3 Association between vital parameters and mood on a momentary level

Associations between vital parameters measured by the move 4 and self-perceived mood ranked on the emoji scale on a momentary level are presented in Table 3. Steps taken during the 60 min before the mood rating were positively associated with self-rated mood (stand. *β* = 0.050; *t* = 2.739; *p* = 0.006). In other words, doing more steps in the time before the mood rating was associated with a higher mood rating, thus supporting hypothesis 2a. In line with hypothesis 2b, time spent in SB in the 60-min timeframe before the mood rating is negatively associated with self-perceived mood (stand. *β* = −0.086; *t* = −4.583; *p* < 0.001). Lastly, interrupted sedentary time from 30 min compared to uninterrupted sedentary bouts is positively related to mood ratings (stand. *β* = 0.063; *t* = 2.992; *p* = 0.003). Hypothesis 2c can be accepted. Time of the day and time of the day squared do have a significant association with mood on a momentary level. The mood was ranked higher on weekends compared to weekdays. As a robustness analysis, we replicated the reported models while including the affective states (i.e., valence, energetic arousal, and calmness) as dependent variables (see Supplementary file S4). Steps taken during the 60 min before the rating were positively associated with valence (stand. *β* = 0.056; *t* = 3.378; *p* < 0.001) and with calmness (stand. *β* = 0.053; *t* = 3.280; *p* = 0.001). More time spent in SB in the timeframe of 60 min is related to lower ratings of valence (stand. *β* = −0.041; *t* = −2.404; *p* = 0.016), energetic arousal (stand. *β* = −0.067; *t* = −3.833; *p* < 0.001) and calmness (stand. *β* = −0.036; *t* = −2.201; *p* = 0.028). There are significant effects for sedentary bouts on energetic arousal (stand. *β* = 0.068; *t* = 3.410; *p* < 0.001), but not for valence or calmness.

**Table 3:** Multilevel analysis for hypothesis 2.

Type of effect	Outcome	Emoji Scale		
	Hypothesis	2a	2b	2c
		b (SE)	b (SE)	b (SE)
Fixed effects	Intercept	5.300 (1.162)**	5.437 (1.161)**	4.931 (1.196)**
	Age (yrs)	−0.010 (0.050)	−0.011 (0.50)	0.003 (0.051)
	BMI (kg/m^2^)	0.030 (0.032)	0.030 (0.032)	0.034 (0.033)
	Sex^1^	0.129 (0.205)	0.130 (0.204)	0.0189 (0.210)
	Time of day	0.209 (0.029)**	0.195 (0.029)**	0.206 (0.031)**
	Time of day squared	−0.007 (0.001)	−0.006 (0.029)**	−0.007 (0.001)**
	Weekend^2^	0.130 (0.051)*	0.120 (0.051)*	0.111 (0.057)
	Steps (60-min)	0.005 (0.002)*	-	-
	Sedentary (60 min)	-	−0.007 (0.002)**	-
	Sedentary bout (30 min) interrupted^3^	-		0.167 (0.056)*

*p < 0.05; **p < 0.001.

1 males compared to females.

2 weekend compared to weekdays.

3 interruptet at least once compared to uninterrupted bouts.

## 4 Discussion

The objective of this study was to examine the relationship between vital parameters derived from wearables and self-rated mood in the everyday lives of adolescents and young adults. On a daily level, steps, standing, and exercise time captured by the Apple Watch was positively associated with daily mood. Although the association revealed small effect sizes, the fact that it was observed a daily level and will be repeatedly experienced by individuals over the study period suggests it could have a meaningful impact on how people feel in response to being physically active. Moreover, exploratory analyses revealed significant associations between self-rated sleep quality and mood. On a momentary level, our data indicated positive associations between steps as well as negative associations between SB and momentary mood ratings.

The relationship between PA, SB, and mood in daily life—examined through AA methods—has become an increasingly growing area of research. The most recent review by Timm et al. identified 66 studies that combined wearable-recorded data with self-reported mood to explore within-subject associations in everyday contexts (Timm et al., 2024). Overall, the review emphasizes that even short bouts of PA, distinct from structured exercise sessions, are positively linked to affective well-being (Timm et al., 2024). However, only a limited number of studies have specifically targeted adolescents and young adults. Our finding—that both a higher number of steps and increased standing time per day are positively associated with mood—aligns with the general conclusions of Timm et al.’s review while also contributing new insights to this underrepresented population (Timm et al., 2024). Previous research focusing on similar age groups has reported comparable results. For instance, Cushing et al. found a positive association between daily step count and overall mood in adolescents (Cushing et al., 2017). Likewise, Ivarsson et al. observed similar effects on positive affect, though they employed a different methodology using self-reported PA ratings (Ivarsson et al., 2021). Consistent with earlier studies (Koch et al., 2020; Yang et al., 2021), our results also revealed associations at the momentary level: specifically, a higher number of steps taken within the 60 min preceding a mood rating was linked to more positive mood scores. These findings suggest that even unstructured daily activities, including standing, may support mood enhancement or help prevent mood decline. This insight could be valuable for designing mental health prevention programs. Future research might also apply compositional reallocation models to further clarify how substituting different movement and non-movement behaviors affects momentary mood (Giurgiu et al., 2022). Several behavioral theories and neurobiological mechanisms may help explain the link between non-exercise PA and mood. For example, a neuroimaging study by Reichert et al. demonstrated differences in subgenual anterior cingulate cortex (sagACC) volume between young adults with higher versus lower levels of non-exercise PA (Reichert et al., 2020). Specifically, individuals with greater sagACC volume exhibited less variability in energy levels, while those with lower non-exercise PA reported significantly lower energy (Reichert et al., 2020). Future studies could explore whether this neurobiological mechanism also applies to adolescents.

Exercise time measured by the Apple Watch was positively associated with mood on a daily level. This suggests that structured exercise sessions during the day are associated with a more positive mood on the same day. These findings align with previous studies (Cushing et al., 2017; Bourke et al., 2023) which summarized, that adolescent’s participation in recreational exercises is associated with positive affect. In contrast, C.-H. Yang et al. reported a significant positive effect from MVPA on affect only for younger individuals (ages 9 to 12; Yang et al., 2021). Therefore, when summarizing the effects of MVPA or exercise on mood dimensions such as valence, energetic arousal, and calmness, overall mixed findings are reported (Bossmann et al., 2013; Koch et al., 2020; Bourke et al., 2021b). However, it should be noted that these results are reported on different levels of analyses (day vs. momentary), which may account for these discrepancies. Moreover, the time point for the assessment of mood may also play a significant role. When moods are assessed during PA or closely after (e.g., by using a PA-triggered design; Ebner-Priemer et al., 2013), they may have different associations compared to random assessment over the day.

On a momentary level, our presented study found negative associations between time spent in SB in the last 60 min before a mood rating as well as sedentary bouts of 30 min uninterrupted sedentary time before a mood rating. These results are in line with previous studies that found significant negative associations between sedentary time and positive affect (Cushing et al., 2017; Yang et al., 2021), valence, or energetic arousal (Giurgiu et al., 2019). In contrast, Kracht and collegues reported no significant associations for positive affect following a 30-min sedentary period (Kracht et al., 2021). However, Kracht et al.’s study population is younger than our analyzed population, and further, the authors reported increasing sedentary time with higher age (Kracht et al., 2021). This idea is also supported by the results of C.-H. Yang et al. who found significantly lower levels of positive affect in adolescents aged 11–13 and lower negative affect in younger children when being sedentary for 15 uninterrupted minutes (Yang et al., 2021). In contrast to Giurgiu et al.’s study, our study found a significant association between more time spent sedentary and calmness (Giurgiu et al., 2019). However, again our sample is on average younger compared to Giurgiu et al.’s study (Giurgiu et al., 2019). Therefore, further studies in a younger aged population are warranted. Several behavioral theories may explain the relationship between SB and mood. For instance, the Theory of Effort Minimization in Physical Activity suggests that individuals naturally prefer the least effortful option when engaging in activities (Cheval and Boisgontier, 2021). In other words, being sedentary requires less effort than maintaining a standing posture or other types of PA, and thus may increase the daily time spent sedentary which in turn may lead to a drop in mood (Giurgiu et al., 2019). Based on these results, the interruption of sedentary time might be crucial for enhancing mood and for feeling energized. Different biological mechanisms (i.e., hormonal regulation or arterial blood flow) may underlie the positive impact of sedentary breaks (Chandrasekaran et al., 2021). Thus, it would be worthwhile to explore the potential benefits of active learning and sedentary breaks in university (Lynch et al., 2022) or school settings, which is currently understudied (Hegarty et al., 2016; McMichan et al., 2018; Amor-Barbosa et al., 2022; Bernal et al., 2023; Contardo Ayala et al., 2024). Following the 24-h physical behavior concept (Pedišić et al., 2017; Rosenberger et al., 2019), the consideration of out-of-school interventions is also recommended (Amor-Barbosa et al., 2022).

In our exploratory analyses, we found no significant associations between heart rate and mood. This finding aligns with the results reported by Simon et al. who observed higher heart rates in various emotional states, including anger, stress, happiness, and anxiety (Simon et al., 2021). These emotions are likely to be situated along different dimensions of the mood scale used in the study. Complementary findings are reported by Hachenberger et al. linking increased heart rate to positive affect in young adults (Hachenberger et al., 2023). Heart rate variability, as used by Hachenberger et al. (2023), can provide an additional perspective on the link between cardiovascular processes and mood. Their findings indicate that lower heart rate variability is associated with higher positive affect when assessed over short time intervals and are in line with earlier laboratory findings (Shi et al., 2017; Hachenberger et al., 2023). Moreover, a promising method for linking heart rate and mood is machine learning approaches (Exler et al., 2016). Sleep duration was also not significantly linked to any of the outcomes investigated. This may be because the association of sleep may not underlie a linear but rather a quadratic or cubic association (Tanihata et al., 2015). Another methodical approach to tackle this problem, used by Collier Villaume et al. (2024), is a categorization into groups with different sleep length (e.g., people who slept less than 7 h are categorized as short sleepers). In addition, sleep health is described as a multidimensional concept by Vorster and colleagues who propose that different dimensions of sleep should not be considered in isolation (for example, more sleep may not be beneficial if sleep quality is poor; Vorster et al., 2024). In particular, a sleep health score comprising different factors of sleep (e.g., duration, efficiency, timing, satisfaction, regularity, disordered sleep, breathing, alertness) might be a promising approach in future research studies. In line with previous studies, self-rated sleep quality is positively associated with mood (Tanihata et al., 2015; Sunderland et al., 2021; Hickman et al., 2024). Therefore, monitoring of sleep quality (either self-reported or derived from trackers) should be acknowledged when developing mobile health solutions to enhance mental health support for adolescents and younger adults.

Several limitations in the presented work should be acknowledged. We used a self-developed emoji scale to monitor the participants’ moods. This scale shows significant positive correlations with an established instrument (Wilhelm and Schoebi, 2007) in our sample. Further analyses of robustness were conducted using the MDBF scale, with the results of these analyses corroborating those obtained through the utilization of the emoji scale. However, we emphasize additional studies addressing validity and reliability, for instance, testing test–retest reliability and indicating potential cultural or age-related differences in emoji interpretation. Due to the limited number of participants, the results should be interpreted with caution, as they may not be representative of the broader population. Additional studies with a bigger sample size or clinical populations (i.e., adolescents with psychological disorders) can lead to further knowledge in this field. Moreover, participants were allowed to choose the time of the questionnaire themselves in a provided timeframe. This self-selection of rating times could introduce potential bias, as participants might preferentially report during certain mood states. However, participants received reminders to rate their mood, but no specific time-triggered prompts. Given the option of triggered prompts to answer them immediately or, i.e., up to 30 min later (for example Bourke et al., 2023) the influence through self-determination is not excluded either way. Future study designs may use a mixed-sampling scheme with both self-selected and random prompt times within the same study to quantify potential differences in reporting patterns. Furthermore, experimental studies with a within-person encouragement design might be a sophisticated approach to gain insights into causal mechanisms of short movement breaks on affective states (Giurgiu et al., 2024). This design is based on micro-randomization of interventions and might be the next step following this observational study. The vital parameters (i.e., exercise, step counts, heart rate, and standing) derived from the Apple Watch are based on built-in sensors and proprietary algorithms. In particular, ongoing changes to algorithms or general software updates prevent control for standardization during the course of study. The measurements captured with Apple devices showed mixed evidence regarding validation performance (Abt et al., 2018; Bai et al., 2018; Fuller et al., 2020; Kwon et al., 2021; Lui et al., 2022). However, commercial devices offer a more user-friendly alternative, as they do not require taping to the body but rather allow for the use of a conventional smartwatch, which can be incorporated into daily life (Mohr et al., 2017; Bai et al., 2018). Furthermore, it cannot be ruled out that contextual factors like social context, screen time, nutrition, cognitive load, or caffeine intake, which have not been investigated, influence the outcomes (Hallgren et al., 2020b; Timm et al., 2024). Those should be considered as additional parameters, but need to be selected carefully to keep the effort for participants as low as possible. Lastly, the associations between mood and PA may underlie a reciprocal nature (Schwerdtfeger et al., 2010). Studies investigated both directions of influence with ambiguous outcomes (Timm et al., 2024). Further studies should take this into account, for example, by applying structural equation modeling.

## 5 Conclusion

Our study identified significant associations between vital parameters—including step count, standing time, exercise duration, and sleep quality—and self-reported mood ratings in a sample of adolescents and young adults, both at the daily and momentary level. Specifically, both unstructured PA (such as standing time and step count) and structured exercise, as measured by the Apple Watch, were positively associated with daily mood ratings. This positive relationship was also evident on a momentary level, with a higher step count in the 60 min preceding a self-reported mood rating linked to more positive mood states. In contrast, more time spent sedentary in the 60 min prior to a mood rating was significantly associated with lower mood, while interrupting SB within the 30 min before a mood assessment was linked to improved mood. Adolescence is a developmental phase often marked by heightened vulnerability to mental health challenges. Given the rising prevalence of mental health disorders in this age group, there is an urgent need for effective and accessible prevention strategies. Our findings suggest that monitoring vital parameters through mobile devices such as smartwatches and smartphones offers a promising, cost-effective, and practical approach for early detection and mental health support. Although there are challenges associated with the use of mobile health technologies, such as potential barriers to implementation or socio-economic access to technology, these approaches to promoting mental wellbeing in adolescents and young adults have considerable potential and should be a key focus for future research. Personalized and user-friendly solutions could be seamlessly integrated into mental health prevention programs, offering an age-appropriate and scalable intervention strategy for this population.

## Data Availability

The raw data supporting the conclusions of this article will be made available by the authors, without undue reservation.

## Footnotes

1
https://smilamind.care/
