# Supplementary material for: Associations between wearables vital parameters and self-perceived mood—an ecological momentary assessment study among healthy adolescents
Source: Front Psychol. 2025 Dec 24;16:1623886. doi: 10.3389/fpsyg.2025.1623886 (PMC12777082; doi:10.3389/fpsyg.2025.1623886)
Supplement: Supplementary file 1 [file Data_Sheet_1.PDF]

S1 Appendix. Equations for the used models.

hypothesis 1a-c

$Y(\text{emoji scale})_{ij} = \gamma_{00} + \gamma_{01} * Age + \gamma_{02} * BMI + \gamma_{03} * Sex + \gamma_{04} * Weekday + \gamma_{10} * \text{step count} + u_{0j} + \varepsilon_{ij}$

$Y(\text{emoji scale})_{ij} = \gamma_{00} + \gamma_{01} * Age + \gamma_{02} * BMI + \gamma_{03} * Sex + \gamma_{04} * Weekday + \gamma_{10} * \text{standing time} + u_{0j} + \varepsilon_{ij}$

$Y(\text{emoji scale})_{ij} = \gamma_{00} + \gamma_{01} * Age + \gamma_{02} * BMI + \gamma_{03} * Sex + \gamma_{04} * Weekday + \gamma_{10} * \text{exercise time} + u_{0j} + \varepsilon_{ij}$

hypothesis 2 a-c:

$Y(\text{emoji scale})_{ij} = \gamma_{00} + \gamma_{01} * Age + \gamma_{02} * BMI + \gamma_{03} * Sex + \gamma_{04} * Weekday + \gamma_{10} * \text{time of day} + \gamma_{20} * \text{time of day}^2 + \gamma_{30} * \text{step count 60min} + u_{0j} + \varepsilon_{ij}$

$Y(\text{emoji scale})_{ij} = \gamma_{00} + \gamma_{01} * Age + \gamma_{02} * BMI + \gamma_{03} * Sex + \gamma_{04} * Weekday + \gamma_{10} * \text{time of day} + \gamma_{20} * \text{time of day}^2 + \gamma_{30} * \text{sedentary time 60min} + u_{0j} + \varepsilon_{ij}$

$Y(\text{emoji scale})_{ij} = \gamma_{00} + \gamma_{01} * Age + \gamma_{02} * BMI + \gamma_{03} * Sex + \gamma_{04} * Weekday + \gamma_{10} * \text{time of day} + \gamma_{20} * \text{time of day}^2 + \gamma_{30} * \text{sedentary bout} + u_{0j} + \varepsilon_{ij}$

- Y_{ij} = *Outcome for Subject j by measurement i*
- γ_{00} = *Global intercept*
- γ_{0k} = *Global slope (k=0-4)*
- u_{0j} = *random intercept*
- γ_{k0} = *subject specific slopes*
- u_{0j} = *subject specific deviation from the slope*
- ε_{ij} = *Level-1 residuals*
