# Supplementary material for: Associations between wearables vital parameters and self-perceived mood—an ecological momentary assessment study among healthy adolescents
Source: Front Psychol. 2025 Dec 24;16:1623886. doi: 10.3389/fpsyg.2025.1623886 (PMC12777082; doi:10.3389/fpsyg.2025.1623886)
Supplement: Supplementary file 2 [file Data_Sheet_2.PDF]

S2 Table. Exploratory analyses

Table 4: Multilevel analysis for explorative analyses

	**Outcome**	**Emoji scale**		
		b (SE)	b (SE)	b (SE)
Fixed effects	Intercept	6,169 (1,203) **	6,421 (1,209) **	6,330 (1,220) **
	Age (yrs)	0,026 (0,052)	0,018 (0,053)	0,020 (0,053)
	BMI (kg/m²)	0,037 (0,033)	0,031 (0,033)	0,034 (0,033)
	Sex[1]	0,200 (0,212)	0,198 (0,213)	0,187 (0,213)
	Weekend[2]	-0,164 (0,065) *	-0,138 (0,066) *	-0,149 (0,067) *
	Heart rate (bpm)	0,011 (0,006)	-	-
	Sleep quality	-	0,003 (0,066) *	-
	Sleep duration (min)	-	-	0,00005 (0,0004)

** p < 0.05; ** p < 0.001*
[1](males compared to females)
[2](weekend compared to weekday)
