# Supplementary material for: Associations between wearables vital parameters and self-perceived mood—an ecological momentary assessment study among healthy adolescents
Source: Front Psychol. 2025 Dec 24;16:1623886. doi: 10.3389/fpsyg.2025.1623886 (PMC12777082; doi:10.3389/fpsyg.2025.1623886)
Supplement: Supplementary file 3 [file Data_Sheet_3.PDF]

S3 Tables. Additional analyses. Additional analyses conducted on day level.

Table 5: Multilevel analysis for additional analyses

	**Outcome**	**valence**		
		b (SE)	b (SE)	b (SE)
Fixed effects	Intercept	88.775 (23.365) **	88.765 (23.367) **	88.887 (23.100) **
	Age (yrs)	-1.109 (1.020)	-1.108 (1.020)	-1.167 (1.009)
	BMI (kg/m²)	0.102 (0.645)	0.102 (0.645)	0.125 (0.638)
	Sex[1]	5.207 (4.118)	5.214 (4.118)	5.394 (4.073)
	Weekend[2]	-1.119 (1.003)	-1.136 (1.014)	-0.610 (1.027)
	Step count	0.0003 (0.0002) *	-	-
	Standing time	-	0.006 (0.003) *	-
	Exercise time	-		0.020 (0.028)

** p < 0.05; ** p < 0.001*
[1](males compared to females)
[2](weekend compared to weekday)

Table 6: Multilevel analysis for additional analyses

	**Outcome**	**energetic arousal**		
		b (SE)	b (SE)	b (SE)
Fixed effects	Intercept	37.106 (25.400)	37.404 (25.399)	88.887 (23.100) **
	Age (yrs)	0.547 (1.108)	0.545 (1.108)	-1.167 (1.009)
	BMI (kg/m²)	0.319 (0.701)	0.318 (0.701)	0.125 (0.638)
	Sex[1]	9.137 (4.477) *	9.133 (4.477) *	5.394 (4.073)
	Weekend[2]	-1.216 (1.223)	-1.561 (1.231)	-0.610 (1.027)
	Step count	0,00004 (0,0001)	-	-
	Standing time	-	0.006 (0.004)	-
	Exercise time	-	-	0.020 (0.028)

** p < 0.05; ** p < 0.001*
[1](males compared to females)
[2](weekend compared to weekday)

Table 7: Multilevel analysis for additional analyses

	**Outcome**	**calmness**		
		b (SE)	b (SE)	b (SE)
Fixed effects	Intercept	90.401 (25.190) **	90.624 (25.188) **	90.479 (24.753) **
	Age (yrs)	-1.328 (1.100)	-1.329 (1.100)	-1.420 (1.081)
	BMI (kg/m²)	0.157 (0.696)	0.0157 (0.696)	0.211 (4.364)
	Sex[1]	4.431 (4.439)	4.427 (4.439)	4.779 (4.364)
	Weekend[2]	-2.039 (1.027) *	-2.289 (1.035) *	-1.861 (1.037)
	Step count	0.00005 (0.0001)	-	-
	Standing time	-	0.003 (0.003)	-
	Exercise time	-	-	-0.017 (0.028)

** p < 0.05; ** p < 0.001*
[1](males compared to females)
[2](weekend compared to weekday)
