# Supplementary material for: Associations between wearables vital parameters and self-perceived mood—an ecological momentary assessment study among healthy adolescents
Source: Front Psychol. 2025 Dec 24;16:1623886. doi: 10.3389/fpsyg.2025.1623886 (PMC12777082; doi:10.3389/fpsyg.2025.1623886)
Supplement: Supplementary file 4 [file Data_Sheet_4.PDF]

S4 Tables. Additional analyses. Additional analyses conducted on a momentary level.

Table 8: Multilevel analysis for additional analyses

	**Outcome**	**valence**		
		b (SE)	b (SE)	b (SE)
Fixed effects	Intercept	83.666 (22.445)**	83.349 (22.450)**	78.438 (22.833)*
	Age (yrs)	-1.461 (0.975)	-1.461 (0.975)	-1.327 (0.990)
	BMI (kg/m²)	0.067 (0.614)	0.068 (0.614)	0.156 (0.625)
	Sex[1]	4.000 (3.963)	4.003 (3.963)	4.808 (4.030)
	Time of day	1.351 (0.417)*	1.411 (0.420)**	1.357 (0.460)*
	Time of day squared	-0.036 (0.015)*	-0.039 (0.015)*	-0.038 (0.017)*
	Weekend[2]	1.284 (0.734)	1.173 (0.735)	1.391 (0.842)
	Steps (60min BA)	0.091 (0.027)**	-	-
	Sedentary (60min BA)	-	-0.055 (0.023)*	-
	Sedentary bout (30min) interrupted[3]	-	-	1.337 (0.823)

** p < 0.05; ** p < 0.001*
[1]males compared to females
[2]weekend compared to weekdays
[3]interruptet at least once compared to uninterrupted bouts

Table 9: Multilevel analysis for additional analyses

	**Outcome**	**energetic arousal**		
		b (SE)	b (SE)	b (SE)
Fixed effects	Intercept	8.233 (24.039)	11.031 (24.039)	-0.341 (23.955)
	Age (yrs)	-0.315 (1.040)	0.327 (1.040)	0.065 (1.033)
	BMI (kg/m²)	0.177 (0.656)	0.174 (0.655)	0.209 (0.653)
	Sex[1]	8.381 (4.235)	8.371 (4.233)	8.109 (4.215)
	Time of day	7.709 (0.539)**	7.403 (0.541)**	7.481 (0.588)**
	Time of day squared	-0.279 (0.019)**	-0.271 (0.019)**	-0.273 (0.021)**
	Weekend[2]	1.246 (0.950)	1.142 (0.947)	2.079 (1.075)
	Steps (60min BA)	0.0566 (0.035)	-	-
	Sedentary (60min BA)	-	-0.113 (0.029)**	-
	Sedentary bout (30min) interrupted[3]	-	-	3.586 (1.052)**

** p < 0.05; ** p < 0.001*
[1]males compared to females
[2]weekend compared to weekdays
[3]interruptet at least once compared to uninterrupted bouts

Table 10: Multilevel analysis for additional analyses

	**Outcome**	**calmness**		
		b (SE)	b (SE)	b (SE)
Fixed effects	Intercept	83.981 (24.908)*	83.552 (24.913)*	80.099 (24.690)*
	Age (yrs)	-1.330 (1.084)	-1.330 (1.084)	-1.280 (1.072)
	BMI (kg/m²)	0.280 (0.682)	0.282 (0.682)	0.306 (0.676)

Sex[1]	4.010 (4.402)	4.014 (4.402)	4.237 (4.360)
Time of day	-0.179 (0.421)	-0.107 (0.425)	0.145 (0.471)
Time of day squared	0.015 (0.015)	0.013 (0.0152)	0.003 (0.017)
Weekend[2]	2.545 (0.743)**	2.437 (0.744)*	2.648 (0.861)*
Steps (60min BA)	0.089 (0.027)*	-	-
Sedentay (60min BA)	-	-0.051 (0.023)*	-
Sedentary bout (30min) interrupted[3]	-	-	0.203 (0.842)

** p < 0.05; ** p < 0.001*

[1]males compared to females

[2]weekend compared to weekdays

[3]interruptet at least once compared to uninterrupted bouts
